# Personalized Management of Hydrosalpinx Before IVF: A Narrative Review of Reproductive Outcomes and Treatment Timing

**DOI:** 10.3390/medicina62101959

**Published:** 2026-10-10

**Authors:** Eduard Cristian Mihoci, Demetra Socolov, Răzvan Vladimir Socolov, Ana Maria Mihoci, Dragos Nemescu

**Affiliations:** 1 Department of Mother and Child Medicine, Grigore T. Popa University of Medicine and Pharmacy, 700115 Iasi, Romania; demetra.socolov@umfiasi.ro (D.S.); razvan.socolov@umfiasi.ro (R.V.S.); craciun.ana-maria@d.umfiasi.ro (A.M.M.); dragos.nemescu@umfiasi.ro (D.N.); 2 Clinical Hospital of Obstetrics and Gynecology “Elena Doamna”, 700398 Iasi, Romania; 3 Clinical Hospital of Obstetrics and Gynecology “Cuza Voda”, 700038 Iasi, Romania

**Keywords:** hydrosalpinx, in vitro fertilization, salpingectomy, proximal tubal occlusion, ovarian reserve, assisted reproductive technology, sclerotherapy, personalized treatment

## Abstract

*Background and Objectives*: Hydrosalpinx is an important and treatable cause of impaired in vitro fertilization (IVF) outcomes, but the optimal intervention and its timing remain uncertain. This narrative review evaluates contemporary evidence to support individualized management before IVF. *Materials and Methods*: Targeted searches of PubMed/MEDLINE records and publisher websites prioritized systematic reviews, meta-analyses, randomized trials, cohort studies, and international guidelines published from January 2015 to September 2026, while retaining earlier landmark studies. Evidence concerning salpingectomy, laparoscopic proximal tubal occlusion (PTO), aspiration, sclerotherapy, ovarian reserve, and treatment timing was synthesized narratively. *Results*: Clinically significant communicating hydrosalpinx should generally be treated before embryo transfer. Salpingectomy remains the most established definitive intervention, whereas laparoscopic PTO appears to provide comparable clinical pregnancy outcomes and may reduce tubo-ovarian vascular disruption in selected patients. Evidence regarding ovarian reserve after salpingectomy is conflicting: total oocyte yield is often preserved, although some studies report lower anti-Müllerian hormone, antral follicle count, or ipsilateral follicular recruitment. Aspiration and sclerotherapy may be considered when standard surgery is not feasible, but recurrence and limited randomized evidence restrict routine use. Observational studies do not establish a universally superior sequence of surgery and oocyte retrieval. *Conclusions*: Management should move from a uniform procedure-centered approach toward patient-centered selection. Reproductive age, ovarian reserve, laterality, pelvic adhesions, surgical complexity, and treatment urgency should guide both the intervention and its timing, while effective hydrosalpinx treatment should precede embryo transfer.

## 1. Introduction

Hydrosalpinx represents an important manifestation of tubal factor infertility and remains clinically relevant in women undergoing assisted reproductive technology (ART). It results from distal tubal occlusion with subsequent accumulation of fluid and tubal distension, most commonly following pelvic inflammatory disease, but also in association with endometriosis, previous pelvic surgery, and adhesive disease [1]. Although in vitro fertilization (IVF) bypasses tubal patency, hydrosalpinx may adversely affect reproductive outcomes even when embryos are transferred directly into the uterine cavity.

The detrimental association between hydrosalpinx and IVF has been recognized for several decades. Early meta-analyses demonstrated lower implantation and pregnancy rates and higher pregnancy loss among women with hydrosalpinx compared with women with other forms of tubal infertility [2,3]. More recent systematic reviews have confirmed the adverse association and supported treatment of clinically significant hydrosalpinx before IVF [1,4,5]. The mechanisms underlying this effect are likely multifactorial and include reflux of hydrosalpingeal fluid into the endometrial cavity, mechanical interference with embryo implantation, chronic inflammation, altered endometrial receptivity, and potentially direct effects of tubal fluid on embryo development [1,5].

Laparoscopic salpingectomy has historically been regarded as the reference treatment before IVF. Randomized evidence indicates that treatment of hydrosalpinx, particularly when visible on transvaginal ultrasound, improves reproductive outcomes [5]. However, complete salpingectomy may not represent the optimal intervention for every patient. Difficult tubo-ovarian anatomy and dense pelvic adhesions may increase operative risk, while dissection of the mesosalpinx has raised concerns regarding possible effects on ovarian vascularization and ovarian reserve [6].

Alternative approaches include laparoscopic proximal tubal occlusion, ultrasound-guided aspiration, and sclerotherapy. Contemporary evidence suggests that proximal tubal occlusion can improve IVF outcomes without requiring removal of the diseased tube, while less-invasive procedures may have a role when standard laparoscopic treatment is not feasible [7]. Importantly, randomized evidence has not established a clear live-birth superiority of one intervention over all others [7].

Current international guidance reflects this uncertainty. The World Health Organization (WHO) guideline published in 2025 conditionally suggests either salpingectomy or tubal occlusion before IVF in women with hydrosalpinx; this recommendation is based on very-low-certainty evidence. The guideline further emphasizes that feasibility, availability of trained providers, and the presence of pelvic adhesions should influence procedure selection [8]. Thus, contemporary management is increasingly shifting from a uniform procedure-based strategy toward individualized treatment.

Treatment timing represents an additional clinical consideration. Surgery is conventionally performed before ovarian stimulation; however, immediate surgery may be less attractive in women with advanced reproductive age or diminished ovarian reserve. In selected patients, ovarian stimulation and oocyte or embryo cryopreservation before hydrosalpinx treatment may preserve reproductive opportunities while allowing definitive treatment before frozen embryo transfer. Recent observational evidence has not demonstrated a clear overall live-birth advantage for either a retrieval-first or surgery-first sequence [9].

The clinically relevant question is therefore no longer simply whether hydrosalpinx should be treated, but which intervention should be selected for which patient and at what point in the IVF pathway. The aim of this narrative review is to provide an updated, clinically oriented synthesis of the evidence regarding salpingectomy, proximal tubal occlusion, aspiration, and sclerotherapy; their potential effects on ovarian reserve; and the timing of intervention relative to IVF. Particular emphasis is placed on integrating reproductive age, ovarian reserve, hydrosalpinx characteristics, pelvic adhesions, and surgical complexity into a practical framework for personalized treatment selection.

## 2. Materials and Methods

### Literature Search Strategy

This narrative review used targeted searches of PubMed/MEDLINE records and publisher websites to identify clinically relevant literature on hydrosalpinx management before IVF. International guidance and the bibliographies of key evidence syntheses provided additional sources. The purpose was a clinically focused synthesis rather than exhaustive retrieval of every eligible study.

The principal search terms included combinations of “hydrosalpinx”, “in vitro fertilization”, “IVF”, “assisted reproductive technology”, “salpingectomy”, “proximal tubal occlusion”, “tubal occlusion”, “ovarian reserve”, “anti-Müllerian hormone”, “AMH”, “antral follicle count”, “aspiration”, “sclerotherapy”, “embryo transfer”, “oocyte retrieval”, and “timing of surgery”.

The literature and key source records were last checked on 9 September 2026. Priority was given to systematic reviews, meta-analyses, randomized trials, cohort studies, and international guidance published from January 2015 to that date. Earlier landmark studies were retained to explain the adverse reproductive association, treatment effects, and proposed mechanisms.

Studies were considered particularly relevant when they reported implantation, clinical pregnancy, ongoing pregnancy, miscarriage, ectopic pregnancy, or live birth outcomes, as well as measures of ovarian reserve or ovarian response to stimulation. Evidence concerning surgical feasibility, complications, pelvic adhesions, and timing of hydrosalpinx treatment relative to ovarian stimulation and embryo transfer was also considered.

Evidence was synthesized by clinical theme: reproductive outcomes, ovarian reserve, procedure selection, and treatment timing. No review protocol was registered, no formal risk-of-bias assessment was performed, and no new meta-analysis was undertaken. Estimates are reported as presented in the cited studies; differences in populations, denominators, and outcome definitions preclude informal comparison across studies. This selective narrative approach is susceptible to selection and publication bias and does not establish the completeness of the evidence base.

Evidence was interpreted according to study design. Randomized controlled trials and systematic reviews/meta-analyses of randomized evidence were given greatest weight for comparative treatment-effect conclusions. Observational studies were used primarily to evaluate ovarian response, treatment timing, surgical feasibility, and outcomes insufficiently addressed by randomized trials, whereas mechanistic studies were used only to discuss biological plausibility. Conclusions derived from observational or mechanistic evidence are identified as such throughout the manuscript.

Clinical pregnancy, ongoing pregnancy, live birth, and cumulative live birth were treated as distinct outcomes and are reported according to the definitions and denominators used in the original studies. Because definitions and follow-up horizons differed across studies, these outcomes were not considered interchangeable.

Generative AI was used as an assistive tool for literature organization, drafting support, language refinement, reference-format checks, and document formatting. The authors defined the clinical question and review scope, selected and critically evaluated the evidence, verified the source material and references, determined the clinical interpretation and synthesis, and approved the final wording and conclusions. All AI-assisted material was reviewed and edited by the authors, who take full responsibility for the accuracy, originality, and integrity of the submitted work.

## 3. Hydrosalpinx and Reproductive Outcomes

### 3.1. Epidemiology and Etiology

Tubal disease accounts for a substantial proportion of female infertility, and hydrosalpinx represents an advanced form of distal tubal pathology. It has been reported in approximately 10–30% of women with tubal factor infertility [10]. Hydrosalpinx develops when distal tubal occlusion prevents normal drainage of tubal secretions, resulting in progressive fluid accumulation and tubal distension.

Previous pelvic inflammatory disease is the most frequent underlying cause, although hydrosalpinx may also occur following pelvic or tubal surgery, in association with endometriosis, or as a consequence of severe pelvic adhesive disease [1,10]. The anatomical presentation is heterogeneous: disease may be unilateral or bilateral, minimally or markedly distended, and isolated or associated with extensive tubo-ovarian adhesions.

This heterogeneity has clinical implications. Even a unilateral communicating hydrosalpinx may expose the uterine cavity to hydrosalpingeal fluid, whereas bilateral disease potentially combines a greater pathological burden with more complex surgical decision-making. Ultrasound visibility also appears relevant because hydrosalpinges detectable by transvaginal ultrasound have historically been associated with particularly unfavorable IVF outcomes [11,12].

### 3.2. Diagnosis and Clinically Relevant Phenotypes

Hydrosalpinx is frequently asymptomatic and may be identified during infertility assessment. Hysterosalpingography can demonstrate distal tubal obstruction and tubal dilatation, whereas transvaginal ultrasound can directly identify sufficiently distended hydrosalpinges. Laparoscopy provides definitive visualization of tubal and pelvic anatomy but is invasive and is generally reserved for patients in whom surgical treatment is planned.

On transvaginal ultrasound, hydrosalpinx typically appears as an elongated or tubular cystic adnexal structure separated from the ovary. Characteristic findings may include incomplete septations, the “waist sign”, and the “beads-on-a-string” appearance associated with chronic tubal disease [13].

The diagnostic performance of transvaginal ultrasound is high when these characteristic features are present. A 2023 systematic review and meta-analysis including six studies and 3974 women reported a pooled sensitivity of 84% (95% CI, 76–89%) and specificity of 99% (95% CI, 98–100%) for hydrosalpinx [13]. Thus, ultrasound is highly specific, although a negative examination does not exclude a non-distended or intermittently visible hydrosalpinx.

Ultrasound visibility may also have prognostic significance. In the study by de Wit et al., ultrasound-visible hydrosalpinges were associated with significantly reduced implantation and ongoing implantation, whereas hydrosalpinges not detectable by ultrasound were not associated with a demonstrable reduction in IVF outcome [11]. Similarly, randomized data from the Scandinavian salpingectomy trial demonstrated the greatest treatment benefit among women with ultrasound-visible disease [12].

These observations should not be interpreted as evidence that smaller hydrosalpinges are clinically irrelevant. Rather, they suggest that hydrosalpinx should be characterized according to clinically meaningful features, including laterality, communication with the uterine cavity, ultrasound visibility, degree of tubal distension, pelvic adhesions, and anticipated surgical complexity.

For the purposes of this review, the term “clinically significant hydrosalpinx” refers primarily to a communicating hydrosalpinx with features suggesting potential exposure of the endometrial cavity to hydrosalpingeal fluid, particularly when ultrasound-visible or substantially distended. This term is used as a clinical descriptor rather than as a formally validated diagnostic category or threshold.

### 3.3. Impact on Implantation, Pregnancy, and Live Birth

The adverse association between untreated hydrosalpinx and IVF outcomes has been demonstrated consistently. Hydrosalpinx is associated with lower implantation and clinical pregnancy rates and with increased risks of pregnancy loss and ectopic pregnancy [1,2,3,4].

A landmark meta-analysis including 5592 women with tubal infertility found a pregnancy rate of 19.7% among women with hydrosalpinx compared with 31.2% among women without hydrosalpinx. Implantation rates were 8.5% and 13.7%, respectively, while delivery rates per embryo transfer were 13.4% versus 23.4% [3]. Although these absolute rates reflect an earlier era of IVF and should not be directly extrapolated to contemporary ART, the direction of the association has remained consistent.

Pregnancy loss is also increased. Harb et al. reported a 74% relative increase in pregnancy loss among women with hydrosalpinx (RR 1.74; 95% CI, 1.43–2.12). Importantly, treatment of hydrosalpinx was associated with a substantially lower risk of pregnancy loss compared with no intervention (RR 0.46; 95% CI, 0.34–0.63) [4].

Randomized surgical evidence further supports a clinically meaningful effect of treatment. In the cumulative analysis of the Scandinavian trial, salpingectomy significantly increased birth rate, with the greatest effect among women with ultrasound-visible hydrosalpinges (HR 3.8; 95% CI, 1.5–9.2) [12].

More recent evidence has refined rather than overturned this conclusion. The 2025 network meta-analysis included nine randomized controlled trials in its primary analysis and found no statistically significant difference in live birth between the various treatment strategies [7]. However, both salpingectomy and laparoscopic proximal tubal occlusion significantly improved clinical pregnancy compared with no treatment [7].

The evidence therefore supports two distinct conclusions. First, clinically significant hydrosalpinx should generally be treated before embryo transfer. Second, the available evidence does not establish that one intervention is universally superior for live birth. This distinction provides the rationale for moving from the question of whether to treat hydrosalpinx toward the more clinically relevant question of how to select the optimal treatment for an individual patient.

## 4. Mechanisms of Impaired IVF Outcomes

The adverse effect of hydrosalpinx on IVF outcome is likely multifactorial. A communicating hydrosalpinx may expose the uterine cavity to tubal fluid, resulting in mechanical interference with implantation, alterations in the endometrial inflammatory environment and receptivity, and potentially direct effects on embryo development. The relative importance of these mechanisms probably varies according to the volume and composition of the fluid and the degree of communication with the uterine cavity.

### 4.1. Mechanical Reflux and Embryo Displacement

Intermittent reflux of hydrosalpingeal fluid into the uterine cavity provides one of the most plausible mechanisms of implantation failure. A distended tube may act as a reservoir from which fluid periodically enters the endometrial cavity and interferes with embryo apposition.

Eytan et al. investigated uterine fluid dynamics in women with hydrosalpinx and proposed that increased tubal pressure generates a fundus-to-cervix pressure gradient that opposes normal uterine transport. Mathematical modeling suggested that the resulting reflux currents could displace transferred embryos away from the implantation site [14]. Although this study involved only five patients and provides mechanistic rather than clinical proof, it offers a plausible explanation for the adverse effect of communicating hydrosalpinx.

The reproductive benefit observed after both salpingectomy and proximal tubal occlusion indirectly supports this hypothesis. Although only salpingectomy removes the diseased tube, both procedures prevent hydrosalpingeal fluid from reaching the uterine cavity. Thus, interruption of tubal–uterine communication itself may represent an important therapeutic mechanism [5,7,8].

### 4.2. Direct Effects on Embryo Development

Direct embryotoxicity has also been proposed, although experimental evidence is inconsistent.

Granot et al. evaluated human tripronuclear embryos exposed to hydrosalpingeal fluid and found no significant impairment of embryonic development at either 50% or 100% fluid concentrations [15]. The authors therefore suggested that mechanical rather than direct cytotoxic effects could be more important in vivo.

Spandorfer et al. found a concentration-dependent effect in murine embryos, but impaired development became apparent mainly at high concentrations. Without endometrial co-culture, inhibition occurred at concentrations of at least 50%, whereas in an endometrial co-culture model designed to more closely approximate the intrauterine environment, impairment was not observed until hydrosalpingeal fluid reached at least 75% of the medium [16].

Together, these findings suggest that direct embryotoxicity is biologically possible but unlikely to represent the sole or universal mechanism responsible for reduced implantation in women with hydrosalpinx.

### 4.3. Inflammation and the Endometrial Microenvironment

Hydrosalpinx frequently results from previous inflammatory tubal injury, and exposure of the uterine cavity to tubal secretions may alter the endometrial immune and molecular environment.

Recent evidence supports this possibility. In a 2025 case–control study involving 15 women with communicating hydrosalpinx and 15 fertile controls, Shamlou et al. demonstrated significantly increased endometrial IL-6 expression and reduced HB-EGF expression during the implantation window. Glycodelin A expression was numerically lower but the difference was not statistically significant [17].

These findings are consistent with altered implantation-related signaling but should be interpreted cautiously because mechanistic studies generally involve small populations and cannot establish that individual molecular abnormalities directly cause IVF failure.

### 4.4. Impaired Endometrial Receptivity

Studies of endometrial receptivity provide biological support for the association between hydrosalpinx and implantation failure.

Meyer et al. evaluated 103 women with hydrosalpinx and demonstrated significantly reduced expression of the αvβ3 integrin compared with infertile and fertile controls. Among 20 women with impaired receptivity who underwent repeat biopsy after treatment of the hydrosalpinx, 70% demonstrated increased αvβ3 expression [18].

HOXA10, a transcription factor involved in endometrial differentiation and implantation, has also been extensively studied. Daftary and Taylor exposed endometrial epithelial cells to hydrosalpingeal fluid obtained from 10 women and demonstrated a concentration-dependent decrease in HOXA10 mRNA expression [19].

Subsequently, Daftary et al. prospectively evaluated nine women with hydrosalpinx before and after salpingectomy. Endometrial HOXA10 expression was reduced before surgery and increased approximately 15-fold following salpingectomy [20]. Although the sample was small, the reversibility of the molecular abnormality provides biologically relevant support for an adverse endometrial effect of hydrosalpinx.

Other receptivity markers appear to be affected. In a prospective study of 20 women with hydrosalpinx and 21 controls, Li et al. found significantly lower endometrial expression of LIF, integrin β3, and MUC1, whereas the proportion and density of pinopodes did not differ significantly [21]. Zhong et al. similarly reported reduced endometrial LIF and L-selectin ligand expression in women with hydrosalpinx, with expression increasing after surgical treatment [22].

More recent findings reinforce this concept. In a 2024 prospective study involving 14 women with communicating hydrosalpinx and 14 fertile controls, Zafarani et al. found reduced expression of several endometrial HOX genes before surgery and postoperative upregulation following salpingectomy, together with changes in HOTAIR expression [23]. The small sample size means that these results should remain mechanistic rather than clinically actionable evidence.

Importantly, none of these molecular markers is currently validated for selecting a hydrosalpinx treatment strategy in routine practice.

### 4.5. An Integrated Mechanistic Model

Current evidence therefore favors an integrated model. Hydrosalpingeal fluid may mechanically interfere with embryo apposition while simultaneously modifying inflammatory signaling and endometrial receptivity; direct effects on embryo development may contribute under some conditions but appear less consistently demonstrated.

This model also helps explain why both salpingectomy and proximal tubal occlusion improve IVF outcomes despite fundamentally different surgical approaches. Salpingectomy removes the diseased tube and fluid reservoir, whereas proximal occlusion leaves the tube in situ but prevents further communication with the uterine cavity.

Therefore, complete removal of the tube may not be biologically necessary to obtain the principal reproductive benefit in every patient. Preventing exposure of the endometrium to hydrosalpingeal fluid may be sufficient in selected women, providing the mechanistic basis for considering proximal tubal occlusion when ovarian reserve or surgical complexity makes salpingectomy less attractive.

## 5. Salpingectomy Before IVF

Laparoscopic salpingectomy remains the most established definitive treatment for hydrosalpinx before IVF. Removal of the affected tube eliminates the hydrosalpingeal fluid reservoir and permanently prevents reflux into the uterine cavity. However, its contemporary role should be considered in relation to disease phenotype, ovarian reserve, and surgical complexity rather than as an obligatory procedure for every patient.

### 5.1. Evidence for Reproductive Benefit

The strongest randomized evidence originates from the Scandinavian multicenter trial by Strandell et al. [24]. In the intention-to-treat analysis of 204 women, clinical pregnancy occurred in 36.6% of patients allocated to salpingectomy compared with 23.9% without intervention, while delivery rates were significantly higher after surgery (28.6% vs. 16.3%; *p* = 0.045).

The benefit was particularly pronounced among women with ultrasound-visible hydrosalpinges. In this subgroup, clinical pregnancy rates were 45.7% after salpingectomy compared with 22.5% without intervention, and delivery rates were 40.0% versus 17.5% [24]. Women with bilateral ultrasound-visible hydrosalpinges experienced an approximately 3.5-fold increase in delivery rate following surgery.

Cumulative follow-up of the randomized population further supported the benefit of intervention [12]. Among 186 women undergoing 452 IVF cycles, salpingectomy was associated with a significantly higher birth rate (HR 2.1; 95% CI, 1.6–3.6). The effect was greater among women with ultrasound-visible hydrosalpinges (HR 3.8; 95% CI, 1.5–9.2), and implantation rates were also significantly higher following surgery [12].

These findings have been supported by subsequent evidence synthesis. Volodarsky-Perel et al. included five randomized and nine observational studies and found that, within randomized studies, salpingectomy was associated with higher live birth (RR 1.59; 95% CI, 1.17–2.16), clinical pregnancy (RR 1.27; 95% CI, 1.02–1.57), and implantation rates (RR 1.55; 95% CI, 1.16–2.08) [25]. Importantly, the number of retrieved oocytes was not significantly reduced.

More recent evidence has refined the interpretation of these results. The 2025 network meta-analysis confirmed favorable effects of salpingectomy compared with no treatment for clinical and ongoing pregnancy but did not demonstrate that salpingectomy was superior to other hydrosalpinx interventions for live birth [7].

Thus, salpingectomy is clearly effective compared with leaving a clinically significant hydrosalpinx untreated, but available evidence does not establish that complete removal of the tube is necessary to obtain the reproductive benefit in every patient.

### 5.2. Surgical Considerations and Patient Selection

Hydrosalpinx commonly occurs in women with previous pelvic inflammatory disease, endometriosis, or pelvic surgery, and the affected tube may be densely adherent to the ovary, bowel, uterus, or pelvic sidewall. Consequently, salpingectomy may vary substantially in technical difficulty.

When surgery is straightforward, dissection should minimize unnecessary injury to the mesosalpinx and ovarian vascular supply, particularly in women undergoing bilateral procedures or those with limited ovarian reserve. The potential relationship between salpingectomy and ovarian reserve is considered separately in Section 6.

Dense tubo-ovarian adhesions may alter the balance between definitive anatomical treatment and surgical morbidity. Extensive adhesiolysis performed solely to remove the complete tube may increase the risk of ovarian, bowel, or vascular injury. In these circumstances, proximal tubal occlusion can achieve the principal reproductive objective of preventing hydrosalpingeal fluid from reaching the uterine cavity while limiting distal tubo-ovarian dissection.

This consideration is consistent with contemporary guidance. WHO conditionally suggests either salpingectomy or tubal occlusion before IVF and specifically identifies feasibility and pelvic adhesions as factors influencing procedure selection [8]. ASRM similarly recognizes both approaches for communicating hydrosalpinges [26].

Laterality is also relevant. A unilateral communicating hydrosalpinx may adversely affect IVF despite a normal contralateral tube and should not automatically be left untreated [26]. However, bilateral salpingectomy exposes both tubo-ovarian vascular territories to surgery and may therefore warrant greater consideration of baseline ovarian reserve.

### 5.3. Salpingectomy Versus a Tubal-Occlusive Strategy

The principal advantage of salpingectomy is its anatomical definitiveness. The pathological tube and its fluid reservoir are removed, eliminating both uterine reflux and the possibility of subsequent enlargement of an isolated distal hydrosalpinx.

However, comparative evidence has not demonstrated consistent superiority over proximal tubal occlusion for IVF outcomes. Meta-analyses of randomized and observational evidence found no significant differences between salpingectomy and proximal occlusion in implantation or clinical pregnancy, while available live-birth evidence remains limited [27,28].

The randomized trial by Vignarajan et al. is particularly relevant because it illustrates the potential trade-off between reproductive effectiveness and ovarian preservation [29]. Implantation, clinical pregnancy, and live-birth rates were not significantly different between salpingectomy and PTO, whereas postoperative AMH and AFC were lower and ovarian stimulation requirements greater after salpingectomy [29].

These findings should not be interpreted as proof that PTO is universally preferable. Rather, they indicate that the additional anatomical benefit of removing the tube must be weighed against operative complexity and potential ovarian consequences in an individual patient. The comparative evidence and ovarian-reserve implications are examined in greater detail in Section 6 and Section 7.

### 5.4. Contemporary Role of Salpingectomy

Salpingectomy should therefore remain a reference treatment within an individualized management strategy.

It is particularly attractive when the hydrosalpinx is readily accessible, ovarian reserve is reassuring, operative risk is low, and definitive removal of the pathological tube provides additional clinical value. The randomized evidence is strongest for large, ultrasound-visible hydrosalpinges [12,24].

The potential ovarian-reserve cost of salpingectomy should be weighed against the longer-term anatomical advantage of definitive removal of the diseased tube. Removal eliminates the hydrosalpingeal reservoir and avoids subsequent enlargement of an isolated distal hydrosalpinx; it may also provide additional clinical value when persistent pain, recurrent infection, marked tubal enlargement, or other clinically relevant tubal pathology is present [26].

Conversely, diminished ovarian reserve, bilateral disease, severe tubo-ovarian adhesions, previous complex pelvic surgery, or extensive anticipated dissection may shift the balance toward proximal tubal occlusion or another individualized strategy.

Procedure selection should weigh the benefit of definitive tubal removal against the ovarian and surgical risks in the individual patient.

## 6. Salpingectomy and Ovarian Reserve

The potential effect of salpingectomy on ovarian reserve remains one of the principal concerns when selecting treatment for hydrosalpinx before IVF. The biological rationale is plausible: tubal and ovarian arterial branches communicate within the mesosalpinx, and extensive coagulation or difficult dissection may theoretically compromise ovarian perfusion. However, the clinical evidence is inconsistent, and changes in biochemical markers do not necessarily translate into reduced oocyte yield or live birth.

### 6.1. Evidence from AMH and AFC

An earlier systematic review and meta-analysis by Mohamed et al. included eight studies evaluating AMH after salpingectomy [30]. In six before-and-after studies involving 464 women, there was no statistically significant postoperative reduction in AMH (WMD −0.10 ng/mL; 95% CI, −0.19 to 0.00). Comparisons with nonsurgical controls were also non-significant. The authors concluded that salpingectomy did not appear to compromise ovarian reserve in the short term, although long-term effects remained uncertain [30].

A broader meta-analysis by Kobayashi et al. subsequently included 21 studies [6]. Longitudinal comparisons of the same women before and after surgery again showed no significant decrease in AMH following either unilateral or bilateral salpingectomy. However, case–control analyses produced a more concerning signal: previous salpingectomy was associated with lower AMH overall (MD −0.31; 95% CI, −0.55 to −0.07), with a larger difference after bilateral surgery (MD −0.71; 95% CI, −1.19 to −0.23). AFC was also lower compared with nonsurgical controls (MD −1.31; 95% CI, −2.13 to −0.48) [6].

These apparently contradictory findings probably reflect methodological differences. Before-and-after studies reduce inter-individual variability but often involve short follow-up and small populations, whereas case–control studies are more susceptible to confounding by the underlying indication for salpingectomy. Women requiring surgery frequently have pelvic inflammatory disease, endometriosis, ectopic pregnancy, or previous pelvic surgery, all of which may independently influence reproductive function.

Therefore, current evidence does not establish that salpingectomy inevitably reduces ovarian reserve, but a modest adverse effect cannot be excluded.

Accordingly, changes in AMH or AFC should be interpreted as changes in ovarian-reserve markers rather than as proof of clinically meaningful reproductive impairment. Their relevance should be considered alongside follicular recruitment, total oocyte yield, embryo outcomes, live birth, cumulative live birth, and the anatomical benefits of definitive tubal removal.

### 6.2. Ovarian Response During IVF

Biochemical markers such as AMH and AFC are surrogate outcomes. For women undergoing IVF, follicular recruitment, oocyte yield, cycle cancellation, embryo availability, and ultimately live birth are more clinically relevant.

Evidence regarding overall ovarian response is relatively reassuring. Yoon et al. evaluated 18 studies including 1482 women and found no significant differences after salpingectomy in peak estradiol, total gonadotropin requirements, or number of retrieved oocytes [31]. Similarly, Volodarsky-Perel et al. found no meaningful reduction in oocyte yield; among randomized studies, the mean difference in retrieved oocytes was −0.03 (95% CI, −0.75 to 0.70) [25].

However, analyses that distinguish the operated ovary from the contralateral ovary suggest that total oocyte yield may conceal localized effects.

Reitz et al. evaluated 24 women undergoing IVF before and after unilateral salpingectomy [32]. Following surgery, fewer mature follicles were observed in the ipsilateral ovary than in the contralateral ovary (3.00 vs. 5.08; *p* = 0.048), although total follicular recruitment was not significantly reduced [32].

This concern has been strengthened by recently published evidence. In 2026, Benchimol et al. compared 114 women with previous unilateral salpingectomy with 228 matched controls [33]. Women with salpingectomy demonstrated a significantly greater inter-ovarian difference in follicular recruitment, consistent with reduced recruitment on the operated side. Nevertheless, the total number of retrieved and mature oocytes was not significantly different.

Of particular interest, cycle cancellation occurred more frequently after salpingectomy (13.2% vs. 3.1%; *p* < 0.001). In the subgroup undergoing salpingectomy specifically for hydrosalpinx, cancellation occurred in 12.5% compared with 4.4% among matched controls [33].

These observations suggest a possible explanation for the apparent discrepancy within the literature: unilateral salpingectomy may reduce ipsilateral follicular recruitment while compensation by the contralateral ovary preserves overall oocyte yield. Whether this compensation is sufficient after bilateral surgery or in women with already diminished ovarian reserve remains uncertain.

### 6.3. Salpingectomy Versus Proximal Tubal Occlusion

Direct comparisons with proximal tubal occlusion are particularly informative because they involve women with the same underlying condition and therefore reduce some of the confounding present when salpingectomy patients are compared with healthy controls.

In the randomized trial by Vignarajan et al., 165 women with hydrosalpinx were assigned to PTO or salpingectomy before IVF [29]. After surgery, AMH was lower following salpingectomy than PTO (2.6 vs. 3.7 ng/mL; *p* < 0.001), as was AFC (8.6 vs. 10.6; *p* < 0.001). Women undergoing salpingectomy also required a higher gonadotropin dose (3901 vs. 3260 IU) and longer stimulation (11.3 vs. 10.2 days) [29].

Despite these differences, implantation, clinical pregnancy, live birth, and miscarriage rates were not significantly different between procedures. Thus, measurable differences in ovarian reserve did not translate into a demonstrated difference in pregnancy outcomes in this trial.

A meta-analysis comparing salpingectomy with PTO similarly favored PTO for AMH and AFC, while FSH did not differ significantly [34]. The 2025 network meta-analysis also identified a greater decrease in AMH following salpingectomy than laparoscopic tubal occlusion, although heterogeneity was substantial [7].

Taken together, these data suggest that PTO may better preserve ovarian reserve markers, but they do not establish superior cumulative reproductive outcomes.

### 6.4. Clinical Interpretation

The available evidence neither establishes a consistent clinically important loss of ovarian reserve nor excludes an adverse effect in vulnerable patients. Importantly, statistically significant changes in AMH, AFC, or ipsilateral follicular recruitment should not be interpreted as clinically meaningful reproductive impairment unless they are accompanied by demonstrable differences in total oocyte yield, embryo outcomes, or cumulative live birth.

At a population level, salpingectomy does not appear to cause a major reduction in total oocyte yield [25,30,31]. Nevertheless, randomized comparisons and more recent ovarian-specific studies indicate that modest reductions in AMH, AFC, or ipsilateral follicular recruitment may occur [6,29,32,33,34].

The clinical significance of this effect is likely to depend on the patient’s baseline reproductive reserve.

In a young woman with reassuring AMH and AFC, unilateral disease, favorable surgical anatomy, and low operative risk, a small potential reduction in ovarian reserve may have limited clinical importance compared with the benefit of definitive treatment.

Conversely, in a woman with advanced reproductive age, diminished ovarian reserve, bilateral hydrosalpinx, previous ovarian surgery, or severe tubo-ovarian adhesions, preservation of ovarian vascularization becomes more important. In such patients, proximal tubal occlusion or, in selected circumstances, oocyte retrieval and embryo cryopreservation before definitive treatment may deserve greater consideration.

Laterality should therefore modify rather than dictate treatment selection. The evidence does not establish an AMH, AFC, or age threshold below which salpingectomy should be avoided.

Most importantly, concern regarding ovarian reserve should not justify leaving a clinically significant communicating hydrosalpinx untreated before embryo transfer. Instead, ovarian reserve should influence how and when the hydrosalpinx is treated.

## 7. Proximal Tubal Occlusion

Laparoscopic proximal tubal occlusion (PTO) represents the principal surgical alternative to salpingectomy for hydrosalpinx before IVF. In this review, PTO refers specifically to laparoscopic interruption of the proximal fallopian tube, thereby disconnecting a communicating hydrosalpinx from the uterine cavity while leaving the distal pathological tube in situ and minimizing dissection of the distal tube and tubo-ovarian interface. The broader term “tubal occlusion” is retained only when reproducing the terminology used by guidelines or source studies that do not specify the proximal laparoscopic technique.

Its biological rationale is directly related to the mechanisms discussed previously. If exposure of the endometrium to hydrosalpingeal fluid is a major determinant of impaired implantation, complete removal of the tube may not be necessary in every patient. This characteristic makes PTO particularly relevant when salpingectomy is technically difficult or preservation of ovarian vascularization is a priority.

### 7.1. Reproductive Effectiveness

Randomized evidence supports PTO as an effective treatment before IVF. Kontoravdis et al. randomized 115 women with unilateral or bilateral hydrosalpinges to proximal tubal occlusion, salpingectomy, or no surgical treatment [35]. PTO significantly improved implantation, clinical pregnancy, and ongoing pregnancy compared with no intervention, while reproductive outcomes were comparable with those following salpingectomy.

Subsequent evidence has confirmed the absence of a clear reproductive advantage of salpingectomy over PTO. A meta-analysis of randomized trials found no significant differences between the procedures in ovarian stimulation duration, number of retrieved or fertilized oocytes, implantation, or clinical pregnancy [27].

Similarly, the 2022 meta-analysis by Ou et al., including eight studies and 716 women, demonstrated no significant differences between salpingectomy and PTO in implantation (OR 1.17; 95% CI, 0.62–2.20), clinical pregnancy (OR 0.82; 95% CI, 0.59–1.15), ongoing pregnancy (OR 0.64; 95% CI, 0.36–1.13), miscarriage, or ectopic pregnancy [28]. Live birth was also not significantly different (OR 0.67; 95% CI, 0.16–2.72), although the wide confidence interval reflects substantial imprecision rather than definitive equivalence.

The 2025 network meta-analysis provides the most contemporary synthesis [7]. Among nine randomized controlled trials, no intervention demonstrated significant superiority for live birth. Laparoscopic tubal occlusion nevertheless achieved the highest SUCRA ranking for this outcome, although rankings should be interpreted cautiously given the limited evidence.

For clinical pregnancy, PTO significantly improved outcomes compared with no treatment (OR 2.55; 95% CI, 1.20–5.51), as did salpingectomy (OR 2.24; 95% CI, 1.30–3.86) [7]. Direct comparison did not demonstrate a significant live-birth difference between the two procedures.

Collectively, these findings support laparoscopic PTO as an evidence-based surgical option rather than merely a rescue procedure when salpingectomy cannot be completed.

### 7.2. Ovarian Reserve and Surgical Complexity

The principal theoretical advantage of PTO is avoidance of extensive mesosalpinx dissection adjacent to the ovary. This may be relevant in women with diminished ovarian reserve, bilateral hydrosalpinx, previous ovarian surgery, or severe tubo-ovarian adhesions.

As discussed in Section 6, the randomized trial by Vignarajan et al. found higher postoperative AMH and AFC and lower ovarian stimulation requirements following PTO than after salpingectomy, while implantation, pregnancy, and live-birth outcomes remained comparable [29]. Meta-analytic evidence similarly suggests greater preservation of AMH and AFC following PTO [34].

These findings do not establish superior cumulative reproductive outcomes for PTO but provide a clinically relevant reason to consider it when even a modest reduction in ovarian response could be important.

Pelvic adhesions provide another important indication. Hydrosalpinx commonly occurs after pelvic inflammatory disease, endometriosis, or previous surgery, and the distal tube may be densely adherent to the ovary, bowel, uterus, or pelvic sidewall. Under these circumstances, complete salpingectomy can require extensive adhesiolysis and increase operative morbidity.

Surrey and Schoolcraft reported comparable ovarian response and IVF outcomes after salpingectomy and PTO and suggested proximal occlusion as an appropriate alternative when dense adhesions make salpingectomy technically difficult [36].

Current guidance supports this approach. The 2025 WHO guideline conditionally suggests either salpingectomy or tubal occlusion before IVF on the basis of very-low-certainty evidence and specifically identifies feasibility, availability of trained providers, and the presence of adhesions as factors that should influence treatment selection [8].

### 7.3. Limitations of Proximal Tubal Occlusion

The principal disadvantage of PTO is that the pathological distal tube remains in situ.

Because the tube is obstructed distally and is additionally disconnected proximally, continued secretion may theoretically lead to enlargement of the residual hydrosalpinx. ASRM recognizes this possibility and notes that distal fenestration may be considered in selected cases when proximal occlusion is performed [26]. However, the incidence and clinical significance of progressive enlargement remain poorly defined.

Retention of the diseased tube may also be undesirable when hydrosalpinx is associated with persistent pelvic pain, recurrent infection, marked enlargement, or suspicious tubal pathology. In such circumstances, removal of the tube provides benefits beyond prevention of fluid reflux and may favor salpingectomy.

Successful PTO also depends on complete interruption of tubal–uterine communication. Incomplete occlusion could allow persistent hydrosalpingeal reflux and undermine the reproductive objective of treatment.

Thus, PTO should not be regarded simply as an easier salpingectomy. It is a distinct therapeutic strategy with a different balance of anatomical definitiveness and preservation of surrounding reproductive structures.

These limitations should be weighed directly against the potential ovarian-reserve and operative advantages of PTO. Neither salpingectomy nor laparoscopic PTO is uniformly superior: the former offers anatomical definitiveness, whereas the latter may reduce distal tubo-ovarian dissection but leaves the pathological tube in situ.

### 7.4. Laparoscopic and Hysteroscopic Occlusion Are Not Equivalent

Evidence supporting laparoscopic PTO should not be extrapolated to historical hysteroscopic occlusion using intratubal devices.

Dreyer et al. randomized 85 women with ultrasound-visible hydrosalpinges to hysteroscopic proximal occlusion using intratubal devices or laparoscopic salpingectomy [37]. Ongoing pregnancy occurred in 26.2% of women following hysteroscopic treatment compared with 55.8% following laparoscopic salpingectomy, demonstrating inferior reproductive outcomes with the hysteroscopic device-based strategy [37].

Similarly, the 2025 network meta-analysis did not demonstrate the same reproductive benefit for intratubal device insertion as for laparoscopic tubal occlusion [7].

Therefore, references to PTO in contemporary management should specify laparoscopic proximal tubal occlusion. Historical hysteroscopic device-based occlusion should not be considered an equivalent alternative.

### 7.5. Contemporary Role of PTO

Current evidence supports laparoscopic PTO as a legitimate alternative to salpingectomy before IVF. Its reproductive effectiveness appears broadly comparable in available studies, while its avoidance of extensive distal tubo-ovarian dissection may be advantageous in selected patients.

No validated AMH, AFC, age, or adhesion threshold currently determines when one strategy becomes superior to the other. Treatment selection should therefore remain individualized according to ovarian reserve, laterality, operative anatomy, symptoms, and surgical risk.

## 8. Aspiration and Sclerotherapy

Although salpingectomy and laparoscopic proximal tubal occlusion remain the best-established interventions for hydrosalpinx before IVF, both require surgery. Ultrasound-guided aspiration and sclerotherapy have therefore been investigated as less-invasive alternatives, particularly when laparoscopy is technically difficult, contraindicated, or associated with disproportionate surgical risk.

The principal advantage of these approaches is avoidance of extensive pelvic and tubo-ovarian dissection. Their main limitation is that the underlying pathological tube remains in situ, making recurrence or reaccumulation of hydrosalpingeal fluid an important consideration.

### 8.1. Ultrasound-Guided Aspiration

Transvaginal ultrasound-guided aspiration involves puncture and drainage of an ultrasound-visible hydrosalpinx, frequently at the time of oocyte retrieval. The procedure temporarily reduces the reservoir of fluid capable of refluxing into the uterine cavity but does not correct distal tubal obstruction or permanently interrupt tubal–uterine communication.

Randomized evidence suggests that aspiration is more effective than leaving hydrosalpinx untreated. Hammadieh et al. randomized 66 women to aspiration during oocyte retrieval or no intervention [38]. Biochemical pregnancy was significantly higher following aspiration (43.8% vs. 20.6%; RR 2.1; 95% CI, 1.02–4.6), while clinical pregnancy was numerically but not significantly higher (31.3% vs. 17.6%). No increase in pelvic infection or ectopic pregnancy was observed.

A subsequent randomized trial involving 110 women with ultrasound-visible hydrosalpinges similarly demonstrated higher implantation and clinical pregnancy rates following aspiration [39]. Importantly, outcomes appeared less favorable when fluid rapidly reaccumulated.

The importance of recurrence was demonstrated more clearly by Fouda et al., who randomized 160 women to aspiration at oocyte retrieval or salpingectomy before IVF [40]. Clinical pregnancy (27.5% vs. 40.0%) and implantation (12.82% vs. 18.95%) numerically favored salpingectomy, although the overall differences were not statistically significant. Rapid fluid reaccumulation occurred in 34.2% of women undergoing aspiration. In this subgroup, both implantation and clinical pregnancy were significantly lower than after salpingectomy, whereas outcomes among women without reaccumulation were not significantly different [40].

Thus, the principal weakness of aspiration is not the drainage procedure itself but its limited anatomical durability.

The 2025 network meta-analysis reached a similar conclusion. In randomized evidence, ultrasound-guided aspiration significantly increased ongoing pregnancy compared with no treatment (OR 2.80; 95% CI, 1.03–7.58), but no clear live-birth benefit was demonstrated [7].

### 8.2. Sclerotherapy

Sclerotherapy attempts to overcome the temporary nature of aspiration by instilling a sclerosant into the hydrosalpinx after drainage. Ethanol has been the most frequently studied agent. The intended effect is damage and fibrosis of the secretory tubal epithelium, thereby reducing subsequent fluid production and recurrence.

Initial prospective evidence was encouraging. Jiang et al. treated 33 women with ultrasound-guided aspiration followed by 98% ethanol sclerotherapy before IVF and reported favorable IVF outcomes without an apparent adverse effect on ovarian reserve or responsiveness [41]. However, the small sample size substantially limits interpretation.

A larger study by Zhang et al. evaluated 157 women treated with 98% ethanol sclerotherapy [42]. Hydrosalpinx recurrence occurred in 34 women (21.7%). Implantation, clinical pregnancy, and live-birth rates were substantially better following sclerotherapy than in women whose hydrosalpinx was left untreated. No significant differences in preterm birth, low birth weight, or congenital abnormalities were identified.

Song et al. subsequently compared IVF outcomes across 482 treatment cycles involving ultrasound-guided sclerotherapy, aspiration alone, or bilateral salpingectomy [43]. Reproductive outcomes after sclerotherapy were not significantly different from those following salpingectomy, whereas aspiration alone produced less favorable implantation and clinical pregnancy rates.

The systematic review and meta-analysis by Cohen et al. included 10 studies [44]. Clinical pregnancy and miscarriage rates were not significantly different between sclerotherapy and salpingectomy in the available comparative evidence. In contrast, aspiration alone was associated with lower clinical pregnancy and higher miscarriage rates than salpingectomy. Recurrence remained an important limitation of both aspiration-based strategies, with rates generally exceeding 20% in the included studies [44].

These results suggest that sclerotherapy may provide a more durable alternative to aspiration alone. However, the evidence base remains predominantly observational, protocols are heterogeneous, and direct randomized comparisons with established laparoscopic treatments are limited.

### 8.3. Contemporary Evidence and Guideline Position

The distinction between randomized and observational evidence is particularly important when interpreting the 2025 network meta-analysis [7].

When analysis was restricted to randomized trials, no hydrosalpinx treatment demonstrated significant superiority for live birth, and randomized live-birth data for sclerotherapy were insufficient. However, in the secondary analysis combining randomized and observational studies, sclerotherapy was associated with significantly higher live birth than no treatment (OR 4.60; 95% CI, 1.21–17.46 in the network estimate) [7].

This result is promising but should not be interpreted as evidence that sclerotherapy is superior to salpingectomy or PTO. The estimate relies substantially on non-randomized evidence and has a wide confidence interval.

WHO 2025 suggests salpingectomy or tubal occlusion rather than transvaginal aspiration before IVF [8]. However, when these surgical approaches are unavailable or not feasible, transvaginal aspiration may be offered. The recommendation is conditional and based on very low-certainty evidence.

Sclerotherapy is not yet supported by evidence of sufficient quality to displace salpingectomy or laparoscopic PTO as a standard first-line treatment.

### 8.4. Role Within Personalized Management

Less-invasive treatment should therefore be considered within a hierarchy of evidence rather than as universally interchangeable with surgical intervention.

Aspiration may be useful when a hydrosalpinx becomes apparent during ovarian stimulation, when surgery cannot reasonably be performed, or as a temporary strategy in selected patients. Its main limitation is rapid fluid reaccumulation.

Sclerotherapy may provide a more durable minimally invasive alternative and avoids mesosalpinx dissection, but current evidence is insufficient to establish equivalence with laparoscopic treatment for live birth.

Salpingectomy or laparoscopic PTO should remain the preferred definitive approaches when surgery is feasible.

For women with severe pelvic adhesions, substantial surgical or anesthetic risk, limited access to laparoscopy, or other circumstances in which conventional surgery is disproportionately burdensome, aspiration or selected sclerotherapy may nevertheless provide clinically reasonable alternatives. They may also be considered when established surgical options are declined after informed counseling, while recognizing their greater risk of recurrence and weaker comparative evidence.

The practical principle is therefore that treatment intensity should reflect both the reproductive importance of eliminating hydrosalpingeal fluid and the morbidity required to achieve that objective.

## 9. Timing of Intervention in Relation to IVF

Although treatment of clinically significant hydrosalpinx before embryo transfer is well established, the optimal sequence of tubal intervention and ovarian stimulation remains uncertain. Traditionally, salpingectomy or proximal tubal occlusion has been performed before initiation of IVF. However, immediate surgery may delay oocyte retrieval and may be less attractive in women with advanced reproductive age or diminished ovarian reserve.

Direct comparative evidence regarding treatment sequence is observational. No randomized trial has established whether hydrosalpinx treatment should universally precede oocyte retrieval. Consequently, the timing of intervention should be distinguished from the more firmly supported principle that clinically significant communicating hydrosalpinx should be treated before embryo transfer.

### 9.1. Treatment First Versus Oocyte Retrieval First

The largest study addressing the sequence of salpingectomy and oocyte retrieval included 1610 women with bilateral hydrosalpinx [45]. Of these, 235 underwent oocyte retrieval before salpingectomy, whereas 1375 underwent salpingectomy before retrieval. Women in the retrieval-first group had higher baseline FSH and lower AMH, indicating important baseline differences and probable selection of women with poorer ovarian reserve for fertility preservation before surgery.

Despite these differences, live birth after the first embryo transfer was not significantly different between the retrieval-first and surgery-first groups (37.7% vs. 36.4%; *p* = 0.717) [45]. No cases of pelvic infection or oocyte or embryo contamination were reported among the 235 women who underwent retrieval before salpingectomy.

A different result was reported in a 2024 retrospective study evaluating proximal tubal occlusion [46]. Among 1490 women, 976 underwent tubal occlusion before controlled ovarian stimulation and 514 underwent oocyte retrieval before occlusion. Occlusion-first treatment was associated with higher clinical pregnancy (55.69% vs. 38.5%), live birth (47.08% vs. 25.26%), and cumulative live birth (69.47% vs. 47.47%) [46].

Conversely, women undergoing retrieval first produced more oocytes (15.10 vs. 13.45) and more available day-3 embryos (10.92 vs. 9.94). No significant differences between sequences were identified among women with diminished ovarian reserve [46].

These findings favor occlusion-first treatment at the population level but must be interpreted cautiously. Treatment allocation was not randomized, and residual confounding may persist despite multivariable adjustment. The absence of a significant difference in the diminished-ovarian-reserve subgroup is particularly relevant because these women are among those for whom immediate fertility preservation may be most valuable.

The most recent evidence provides further support for individualization. Wang et al. evaluated 377 women undergoing salpingectomy and IVF/ICSI [9]. Oocyte retrieval preceded salpingectomy in 190 women, whereas 187 underwent surgery first. Observed live-birth proportions were similar between strategies (52.1% vs. 52.9%), with no statistically significant difference [9]. Surgery first was associated with lower time and economic costs and fewer complications after oocyte retrieval; these associations remain susceptible to selection bias and local practice effects.

Taken together, these observational studies do not establish a universally superior sequence. Treatment first appears appropriate for most women, whereas retrieval first remains a reasonable option when delaying ovarian stimulation may carry clinically meaningful reproductive consequences.

### 9.2. How Long Should IVF Be Delayed After Treatment?

The available evidence does not support an arbitrary prolonged waiting interval after successful hydrosalpinx treatment.

In the 2023 bilateral-salpingectomy study, frozen embryo transfer performed within 3 months of surgery produced similar clinical pregnancy and live-birth rates to transfer performed later. Clinical pregnancy was 39.0% within 3 months and 38.5% after 3 months, while live birth was 37.7% and 38.5%, respectively [45].

The interval between surgery and ovarian stimulation is less clear. In the same study, adjusted cumulative pregnancy and live-birth outcomes were higher when oocyte retrieval occurred 4–6 or 7–12 months after salpingectomy than within 3 months [45]. However, women with longer intervals differed in age and ovarian reserve, and fewer oocytes were retrieved with increasing delay, making a causal interpretation difficult.

A 2025 study specifically investigated timing after proximal tubal occlusion in 976 women [47]. Increasing delay was associated with lower clinical pregnancy (adjusted OR 0.904; *p* = 0.001) and live birth (adjusted OR 0.926; *p* = 0.010). Cumulative live-birth rates were 76.34% when retrieval occurred within 3 months, 71.53% at 3–7 months, and 58.77% after more than 7 months [47]. This association does not establish a causal waiting-time threshold or justify delaying treatment to a particular interval.

However, an important methodological issue limits interpretation. The 976 women in this 2025 analysis were treated at the same reproductive center during January 2016–December 2022, corresponding to the center, study period, and exact size of the occlusion-first population in the 2024 report [46,47]. The clinical datasets therefore appear to overlap substantially and should not be interpreted as independent confirmation.

The 2026 salpingectomy study also failed to identify a clearly superior embryo-transfer interval after surgery [9].

Current evidence therefore does not justify a universal rule requiring women to wait 3, 6, or another predetermined number of months after hydrosalpinx treatment. IVF can generally proceed after adequate postoperative recovery, provided that treatment has been effective and no surgical complication requires further delay.

### 9.3. When May Retrieval Before Treatment Be Reasonable?

The principal argument for oocyte retrieval before hydrosalpinx treatment is preservation of current reproductive potential.

For a young woman with reassuring AMH and AFC, a short delay for salpingectomy or PTO is unlikely to meaningfully affect the probability of obtaining oocytes. Treatment first also simplifies the pathway by avoiding mandatory cryopreservation solely because tubal surgery remains pending.

The balance may differ in women with advanced reproductive age, diminished ovarian reserve, previous ovarian surgery, bilateral hydrosalpinx, or anticipated technically difficult surgery. In these circumstances, even a modest delay or reduction in ovarian response may carry greater clinical consequences.

Available evidence provides some reassurance regarding the safety of this strategy. No pelvic infection or oocyte/embryo contamination occurred among the 235 retrieval-first patients in the 2023 study [45]. Nevertheless, this retrospective observation should not be interpreted as proof that retrieval in the presence of hydrosalpinx is risk-free. Tubo-ovarian anatomy, previous pelvic infection, accessibility of the ovaries, and the anticipated needle trajectory should be considered individually.

Most importantly, retrieval-first management should not be confused with leaving hydrosalpinx untreated. It changes the sequence of treatment while maintaining treatment before embryo transfer.

### 9.4. Practical Timing Strategy

For most women, salpingectomy or laparoscopic PTO followed by recovery and IVF is a practical sequence. Retrieval and cryopreservation before surgery may be considered when delay or difficult surgery threatens current reproductive opportunities, with treatment completed before frozen embryo transfer. Both pathways require individualized counseling; comparative evidence is observational [9,45,46,47].

Evidence is insufficient to define an AMH, AFC, age, or postoperative interval that mandates one pathway over the other.

## 10. A Personalized Management Framework

Current evidence supports treatment of clinically significant hydrosalpinx before embryo transfer but does not identify a single intervention as optimal for every patient. Treatment selection should therefore balance the reproductive benefit of eliminating hydrosalpingeal exposure to the uterine cavity against ovarian reserve, reproductive age, disease laterality, surgical complexity, and the urgency of fertility treatment.

Both WHO and ASRM recognize salpingectomy and proximal tubal occlusion as appropriate approaches before IVF [8,26]. Furthermore, contemporary randomized evidence does not demonstrate clear live-birth superiority of salpingectomy over laparoscopic tubal occlusion [7]. These findings provide the rationale for a patient-centered rather than procedure-centered strategy.

### 10.1. Step 1: Define the Clinically Relevant Hydrosalpinx

The first step is to characterize the disease rather than treating hydrosalpinx as a binary diagnosis.

Particular attention should be given to:

Unilateral versus bilateral disease;

Communication with the uterine cavity;

Ultrasound visibility and degree of tubal distension;

Associated pain or other tubal pathology;

Previous pelvic surgery or endometriosis;

Anticipated tubo-ovarian adhesions and operative complexity.

Ultrasound-visible and communicating hydrosalpinges have been associated with the clearest adverse effect on IVF outcomes and the greatest demonstrated benefit from intervention [11,12,24].

Once a clinically significant communicating hydrosalpinx has been identified, treatment before embryo transfer should generally be planned.

### 10.2. Step 2: Integrate Ovarian Reserve and Surgical Complexity

The choice between salpingectomy and laparoscopic PTO should then incorporate reproductive vulnerability and operative anatomy.

Salpingectomy is particularly appropriate when the affected tube is readily accessible, ovarian reserve is reassuring, and removal can be performed without extensive tubo-ovarian dissection. It provides definitive anatomical treatment and has the longest-established evidence base.

The balance may shift toward laparoscopic PTO when ovarian reserve is diminished, disease is bilateral, previous ovarian surgery has occurred, or dense adhesions make salpingectomy technically difficult. Evidence suggests that AMH, AFC, or ipsilateral follicular recruitment may be lower following salpingectomy in some populations, although total oocyte yield is often preserved [6,29,33,34].

Similarly, extensive adhesiolysis solely to achieve complete salpingectomy may add surgical morbidity without proven additional reproductive benefit over effective proximal occlusion.

No validated AMH, AFC, age, or adhesion threshold currently determines when PTO should replace salpingectomy. These factors should therefore modify rather than dictate treatment selection.

### 10.3. Step 3: Individualize the Sequence of IVF and Tubal Treatment

For most women with reassuring ovarian reserve and no urgent reason to initiate stimulation, treatment first remains the simplest pathway:

hydrosalpinx treatment → ovarian stimulation → oocyte retrieval → embryo transfer.

However, selected women with advanced reproductive age, diminished ovarian reserve, previous ovarian surgery, bilateral disease, or anticipated treatment delay may reasonably undergo:

ovarian stimulation → oocyte retrieval and embryo cryopreservation → hydrosalpinx treatment → frozen embryo transfer.

Current evidence comparing these sequences is observational and inconsistent [9,45,46,47]. Therefore, retrieval-first management should not be considered superior to treatment-first management, but neither should it be excluded when immediate preservation of reproductive potential is clinically important.

The stronger and more consistent principle concerns embryo transfer: clinically significant communicating hydrosalpinx should be treated before transfer.

### 10.4. Step 4: Reserve Less-Invasive Approaches for Selected Patients

When laparoscopy is contraindicated, unavailable, or associated with disproportionate operative risk, ultrasound-guided aspiration may be considered [8]. However, fluid reaccumulation reduces its reliability [38,39,40].

Sclerotherapy may provide a more durable minimally invasive alternative and has produced promising reproductive outcomes [41,42,43,44]. Nevertheless, the limited randomized evidence prevents its routine positioning alongside salpingectomy or laparoscopic PTO as an established first-line treatment.

Accordingly, aspiration and sclerotherapy are best regarded as individualized alternatives when standard surgical treatment is not reasonably feasible, is contraindicated, or is declined after informed discussion; they should not be considered directly equivalent to salpingectomy or laparoscopic PTO.

### 10.5. Proposed Clinical Decision Framework

The main management options for hydrosalpinx before IVF are summarized in Table 1.

This framework is an evidence-informed clinical synthesis, not a validated decision rule or formal guideline. Several branches have not been tested in direct randomized comparisons, and no validated ovarian-reserve threshold currently identifies the procedure or treatment sequence that maximizes cumulative live birth for an individual patient.

The choice of procedure and treatment sequence should be discussed with the patient, including surgical morbidity, uncertainty about ovarian effects, and the consequences of bilateral tubal treatment for spontaneous conception [8,26].

## 11. Current Recommendations, Evidence Gaps, and Future Directions

Contemporary recommendations consistently support treatment of clinically significant hydrosalpinx before IVF, but provide substantially less certainty regarding the optimal intervention for an individual patient. ASRM recognizes both laparoscopic salpingectomy and proximal tubal occlusion as effective approaches for communicating hydrosalpinges before IVF [26]. Similarly, the 2025 WHO guideline conditionally suggests either salpingectomy or tubal occlusion before IVF and recommends that feasibility, availability of trained providers, and pelvic adhesions be considered when selecting the procedure [8]. Importantly, this WHO recommendation is based on very-low-certainty evidence.

This uncertainty is reflected in contemporary comparative evidence. The 2025 network meta-analysis found that salpingectomy and laparoscopic tubal occlusion significantly improved clinical pregnancy compared with no treatment, but randomized evidence did not demonstrate significant differences between treatment strategies for live birth [7]. Thus, the evidence supporting treatment of hydrosalpinx is stronger than the evidence supporting one universally superior treatment.

Several clinically important evidence gaps remain.

First, adequately powered randomized trials comparing salpingectomy directly with laparoscopic PTO are needed, with live birth and cumulative live birth as primary outcomes. Ovarian response, cycle cancellation, complications, treatment delay, and cumulative oocyte and embryo yield should also be evaluated.

Second, comparative studies should stratify patients according to reproductive vulnerability. No validated age, AMH, or AFC threshold currently identifies women in whom PTO should be preferred to salpingectomy. This is particularly important because the clinical consequences of a modest change in ovarian response are unlikely to be equivalent in a young woman with reassuring ovarian reserve and a woman of advanced reproductive age with diminished reserve.

Third, the optimal sequence of tubal treatment and oocyte retrieval remains uncertain. Available studies are observational and provide conflicting results [9,45,46,47]. Randomized or well-designed prospective studies are needed to determine whether selected women with diminished ovarian reserve or advanced reproductive age benefit from oocyte retrieval and cryopreservation before hydrosalpinx treatment.

Fourth, the long-term ovarian consequences of salpingectomy require further investigation. Total oocyte yield is generally preserved, but recent evidence suggests that ipsilateral follicular recruitment may be reduced following unilateral salpingectomy [33]. These ovarian-response findings should be distinguished from embryo outcomes and from cumulative live birth, for which clinically meaningful impairment has not been consistently demonstrated. Future studies should therefore evaluate ovarian-specific responses and cumulative reproductive outcomes rather than relying exclusively on serum AMH or AFC.

Fifth, less-invasive approaches require stronger comparative evidence. Aspiration is limited by fluid reaccumulation [38,39,40], while sclerotherapy has produced encouraging results but remains supported largely by observational evidence [41,42,43,44]. Standardized treatment protocols and randomized comparisons with laparoscopic procedures are needed before sclerotherapy can be considered an established first-line alternative.

Future research should therefore move beyond the question of which procedure is globally “best” and instead determine which intervention and treatment sequence are most appropriate for specific patient profiles. Reproductive age, ovarian reserve, hydrosalpinx phenotype, surgical complexity, treatment delay, cost, complications, patient preferences, and cumulative live-birth probability should be integrated into future prospective studies.

Until such evidence becomes available, individualized management remains a reasonable interpretation of the existing literature. Selected studies and their principal limitations are summarized in Table 2.

## 12. Conclusions

Hydrosalpinx is an important and treatable cause of impaired IVF outcome. Its adverse reproductive effect appears multifactorial, involving reflux of hydrosalpingeal fluid into the uterine cavity, mechanical interference with implantation, altered inflammatory signaling, and impaired endometrial receptivity.

Treatment of clinically significant communicating hydrosalpinx before embryo transfer should remain a central principle of IVF management. Laparoscopic salpingectomy provides definitive treatment and has the longest-established evidence base. However, it should not automatically be considered the optimal procedure for every patient. Laparoscopic proximal tubal occlusion provides an effective alternative, particularly when preservation of the tubo-ovarian vascular interface or avoidance of extensive pelvic dissection is important.

The possible effect of salpingectomy on ovarian reserve appears modest and heterogeneous at the population level, but its clinical importance may increase in women with advanced reproductive age, diminished ovarian reserve, bilateral disease, previous ovarian surgery, or technically difficult pelvic anatomy.

Similarly, treatment timing should be individualized. For most women with adequate ovarian reserve, treatment before ovarian stimulation remains the simplest pathway. In selected women with time-sensitive reproductive potential, oocyte retrieval and cryopreservation before hydrosalpinx treatment may be considered, provided that the hydrosalpinx is treated before embryo transfer.

Aspiration and sclerotherapy remain less-invasive alternatives when standard surgical treatment is not reasonably feasible, although recurrence and limited high-quality evidence restrict their routine use.

Prospective comparative studies stratified by ovarian reserve and surgical complexity are needed to determine whether this individualized approach improves cumulative live birth.

A practical evidence-informed pathway can be summarized in six steps:

1. Confirm that the hydrosalpinx is clinically relevant, particularly whether it communicates with the uterine cavity and is ultrasound-visible or substantially distended.
2. Plan effective treatment before embryo transfer.
3. Assess reproductive age, ovarian reserve, laterality, symptoms, previous ovarian or pelvic surgery, adhesions, and anticipated surgical complexity.
4. Favor salpingectomy when definitive removal is technically straightforward and clinically advantageous; consider laparoscopic PTO when avoiding extensive tubo-ovarian dissection is important.
5. Reserve aspiration or selected sclerotherapy for patients in whom established surgical options are not reasonably feasible, are contraindicated, or are declined after informed counseling.
6. Individualize whether oocyte retrieval should precede tubal treatment in women with time-sensitive reproductive potential, while ensuring that the hydrosalpinx is treated before embryo transfer.

This pathway is a clinical synthesis of the available evidence and should not be interpreted as a validated decision rule or formal guideline.

## Figures and Tables

**Table 1:** Evidence-informed management options for hydrosalpinx before IVF.

Clinical Scenario	Suggested Strategy	Rationale and Supporting References
Reassuring ovarian reserve and technically accessible hydrosalpinx	Laparoscopic salpingectomy	Definitive treatment with established reproductive benefit [5,8,24,26]
Diminished ovarian reserve or advanced reproductive age	Individualized salpingectomy versus laparoscopic PTO	Greater importance of minimizing potential ovarian vascular disruption [6,8,29,33]
Bilateral hydrosalpinx with limited ovarian reserve	Consider laparoscopic PTO	Avoid bilateral distal tubo-ovarian dissection [8,29,34]
Severe tubo-ovarian or pelvic adhesions	Consider laparoscopic PTO	Achieve uterine isolation while reducing extensive adhesiolysis [8,26,36]
Hydrosalpinx associated with pain, marked enlargement, recurrent infection, or additional tubal pathology	Salpingectomy when technically feasible	Removal provides benefit beyond prevention of uterine reflux [10,26]
Advanced reproductive age or diminished reserve with clinically important concern regarding treatment delay	Consider retrieval/cryopreservation before tubal treatment	Preserve current reproductive potential; treat before FET [9,45,46,47]
Laparoscopy contraindicated or not reasonably feasible	Ultrasound-guided aspiration ± selected sclerotherapy	Less-invasive alternative, recognizing recurrence and weaker evidence [8,40,44]
Rapid reaccumulation after aspiration	Definitive salpingectomy or laparoscopic PTO before embryo transfer	Recurrent fluid may restore the adverse intrauterine environment [26,40]

PTO, laparoscopic proximal tubal occlusion; FET, frozen embryo transfer. Options reflect clinical synthesis, not validated subgroup-specific treatment effects.

**Table 2:** Selected evidence informing procedure choice and treatment timing.

Source and Design	Main Clinical Finding	Interpretation and Limitations
Strandell et al. [12,24] Randomized trial and cumulative follow-up	Salpingectomy improved delivery outcomes; benefit was greatest with ultrasound-visible disease.	Historical IVF practice; subgroup effects and crossover limit extrapolation.
Melo et al. [5] Cochrane systematic review	Surgical treatment supports improved clinical pregnancy before IVF.	Live-birth evidence and direct comparisons remain limited.
Pérez-Milán et al. [7] Network meta-analysis	Salpingectomy and laparoscopic occlusion improved clinical pregnancy versus no treatment.	Nine RCTs in the primary analysis; no significant live-birth superiority.
Vignarajan et al. [29] Randomized trial, 165 women	Ovarian reserve markers favored PTO; live-birth differences were not significant.	Surrogate differences do not demonstrate greater cumulative reproductive benefit.
Benchimol et al. [33] Matched retrospective study	Ipsilateral recruitment and cycle cancellation raised concern; total oocyte yield was similar.	Unilateral surgery; included ectopic pregnancy and hydrosalpinx; residual confounding.
He et al.; Li et al.; Wang et al. [9,45,46,47] Retrospective cohorts	Results for surgery-first versus retrieval-first pathways were inconsistent.	Selection bias; populations in [46,47] appear to overlap. No causal waiting interval established.

RCT, randomized controlled trial; PTO, laparoscopic proximal tubal occlusion. This table is a selective evidence summary, not an independent systematic review.

## Data Availability

No new data were created or analyzed in this study. Data sharing is not applicable to this article.

## Abbreviations

AFC, antral follicle count; AMH, anti-Müllerian hormone; ART, assisted reproductive technology; ASRM, American Society for Reproductive Medicine; CI, confidence interval; FET, frozen embryo transfer; FSH, follicle-stimulating hormone; HR, hazard ratio; ICSI, intracytoplasmic sperm injection; IVF, in vitro fertilization; LIF, leukemia inhibitory factor; MD, mean difference; OR, odds ratio; PTO, laparoscopic proximal tubal occlusion; RR, risk ratio; SUCRA, surface under the cumulative ranking curve; WHO, World Health Organization; WMD, weighted mean difference.
